# *Oldenlandia diffusa* Extract Inhibits Biofilm Formation by *Haemophilus influenzae* Clinical Isolates

**DOI:** 10.1371/journal.pone.0167335

**Published:** 2016-11-30

**Authors:** Takeaki Wajima, Yui Anzai, Tetsuya Yamada, Hideaki Ikoshi, Norihisa Noguchi

**Affiliations:** 1 Department of Microbiology, School of Pharmacy, Tokyo University of Pharmacy and Life Sciences, Tokyo, Japan; 2 Department of Traditional Chinese Medicine, School of Pharmacy, Tokyo University of Pharmacy and Life Sciences, Tokyo, Japan; Laurentian, CANADA

## Abstract

*Oldenlandia diffusa* has been empirically used as a therapeutic adjunct for the treatment of respiratory infections. To establish the basic evidence of its clinical usefulness, antimicrobial and biofilm inhibitory activities of an *O*. *diffusa* extract were examined against clinical isolates of *Haemophilus influenzae*, a major causative pathogen of respiratory and sensory organ infections. No significant growth inhibitory activity was observed during incubation for more than 6 h after the extract addition into a culture of *H*. *influenzae*. On the other hand, biofilm formation by *H*. *influenzae*, evaluated by a crystal violet method, was significantly and dose-dependently inhibited by the *O*. *diffusa* extract. Furthermore, the mRNA level of the biofilm-associated gene *luxS* of *H*. *influenzae* significantly decreased soon after the extract addition, and the suppressive effect continued for at least 2 h. At 2 h after the addition of the *O*. *diffusa* extract, the autoinducer in the culture supernatant was also significantly reduced by the *O*. *diffusa* extract in a dose-dependent manner. These results revealed that *O*. *diffusa* extract shows inhibitory activity against *luxS-*dependent biofilm formation but has no antimicrobial activity against planktonic cells of *H*. *influenzae*. Thus, *O*. *diffusa* extract might be useful as an adjunctive therapy for the treatment of respiratory infections caused by *H*. *influenzae*.

## Introduction

In recent years, antimicrobial-resistant pathogens have become a concern for respiratory and sensory organ infections [1]. However, development of novel agents has decreased, and physicians have to deal with existing agents. Alternative complementary medicine, including traditional Chinese medicine, has attracted attention as drug therapy that does not rely on antimicrobial agents. Several traditional Chinese medicines have been empirically used for the treatment of infectious diseases, while the basic scientific evidence of their usefulness is lacking. Among traditional Chinese medicines, *Oldenlandia diffusa* (Odi) has been used for inflammatory and infectious diseases, such as pneumonia, appendicitis, and urinary tract infections, as an herb that clears heat and relieves toxicity [2]. Odi has been empirically administered (12–24 g/day orally) for respiratory infections, along with anti-infective medicines. Moreover, Odi has been reported to also show anticancer and immunomodulating activities [3, 4].

*Haemophilus influenzae* and *Streptococcus pneumoniae* are the major causative bacterial agents of respiratory and sensory organ infections. *H*. *influenzae* type b and *S*. *pneumoniae* infections have decreased due to routine vaccination (Hib and pneumococcal conjugate vaccines), whereas the percentage of nontypeable *H*. *influenzae* infections is on the rise [5, 6]. Among respiratory infections caused by *H*. *influenzae*, bronchitis is known to be prone to severe complications [7]. Otitis media and sinusitis, which are sensory organ infections caused by *H*. *influenzae*, can become chronic and intractable in children [7]. These intractable infections may be associated with antimicrobial resistance and biofilm formation by the bacteria [8]. Thus, inhibition of biofilm formation has been suggested to be important for preventing chronic and intractable infections. Biofilm formation by *H*. *influenzae* is known to be associated with quorum sensing (QS) via the LuxS autoinducer system and/or QS two-component control system QseBC [9, 10]. Therefore, inhibition of these systems can result in the inhibition of biofilm formation.

In this study, to establish the basic evidence of the usefulness of Odi extract (OdiE) against infections, we analyzed growth and biofilm inhibitory effects of OdiE on clinical isolates of *H*. *influenzae*.

## Materials and Methods

### Bacterial strains, culture conditions, and chemicals

A total of 20 *H*. *influenzae* strains were randomly selected among clinical isolates obtained at the Tokyo Medical University Hachioji Medial Center between 2011 and 2013 [11]. *H*. *influenzae* ATCC 49247 purchased from the American Type Culture Collection (ATCC; Manassas, VA, USA) was used as a reference strain. These strains were cultured at 35°C under ambient air on chocolate agar or in brain heart infusion broth supplemented with 10 μg/mL NAD and 10 μg/mL hemin (sBHI broth).

For the autoinducer (AI)-2 bioassay, we used *Vibrio harveyi* ATCC BAA-211 purchased from ATCC. This strain was cultured at 30°C in AI bioassay (AB) medium [10], [12].

Haku ka ja zetu sou (Iskra Industry Co., Ltd., Tokyo, Japan), which is sold as a healthy food in Japan, was used as the OdiE.

### Measurement of growth inhibitory activity

Overnight cultures of the test strain were diluted with sBHI broth (1:100) in the presence or absence of OdiE and incubated at 35°C with shaking. The cultures were sampled at 0, 1, 2, 4, 6, 8, 12, and 24 h of incubation, and the samples were diluted with saline. The dilutions were spread on chocolate agar plates and cultured at 35°C overnight. Thereafter, the number of grown colonies was counted to calculate the number of bacterial CFU/mL in an undiluted broth culture. All experiments were performed at least three times on separate days.

### Biofilm formation assay

Biofilm formation was evaluated by the crystal violet assay as reported previously [13]. Briefly, *H*. *influenzae* was cultured overnight in sBHI broth and diluted 1:100 in fresh sBHI. This suspension (100 μL) was transferred into a 96-well microtiter plate (Iwaki, Tokyo, Japan) and cultured for 24 h in the presence or absence of OdiE (2.5, 5, 10, and 20 mg/mL) at 35°C. Then, each well was washed three times with phosphate-buffered saline (PBS) to remove floating bacterial cells. The biofilms were stained for 20 min with 0.1% crystal violet and washed three times with PBS. The remaining crystal violet was dissolved with 200 μL of 95% ethanol, and the absorbance (630 nm) was measured in each well. The test was carried out using five wells per each assay and at least three times on independent occasions.

To evaluate its degradation activity on mature biofilm, OdiE was added to the biofilm formed as described above, and the plate was incubated for 3, 6, and 24 h at 35°C. The remaining biofilm was measured by staining with crystal violet as described above.

### Semi-quantitative reverse transcription–PCR

To compare mRNA levels of biofilm-associated genes (*luxS* and *qseC*), we performed semi-quantitative reverse transcription (RT)–PCR. Cultures were sampled at 0, 1, 2, 4, and 6 h after the addition of OdiE (20 mg/mL). Then, total RNA was extracted from the culture using a High Pure RNA isolation kit (Roche Diagnostics, Tokyo, Japan). PCR was performed as described previously [14]. GyrB-F (GGAAAATCCTGCAGATGC), GyrB-R (AAGCAACGTACGGATGTG), luxS-F (AAAAATGAACGCACCTGCAG), luxS-R (GTACACCTAAAACATCTTGC), qseC-F (TTAAATCCGTGTAATTCCGC), and qseC-R (TGAGCGTTATTTTGTGGCAG) were used as the primers. The resulting PCR products were separated by electrophoresis, and densitometric analysis was performed using the ImageJ software (http://imagej.nih.gov/ij/). The transcriptional level of *gyrB* was used as an internal control.

### Autoinducer bioassay

*H*. *influenzae* was cultured in 10 mL of sBHI overnight. Bacterial cells were centrifuged and resuspended in 10 mL of fresh sBHI to avoid the carryover of AI. The resultant suspension was diluted 10-fold in sBHI and cultured with shaking for 2 h after the addition of OdiE. Then, the bacterial cells were removed by centrifugation and filtration (pore size 0.45 μm).

*V*. *harveyi* ATCC BAA-211 was cultured in AB medium overnight and diluted 1:5,000 in fresh AB medium. Aliquots (100 μL) of this suspension were transferred into a black 96-well plate (STEM, Tokyo, Japan) and mixed with 10 μL of an *H*. *influenzae* supernatant diluted 1:2 with fresh BHI. The plate was incubated for 5 h at 30°C, and the bioluminescence signal was measured. *V*. *harveyi* incubated in BHI was included as a background control. All experiments were performed at least twice on separate days.

### Statistical analysis

Statistical differences were assessed by Student's and Welch's *t*-tests using the JMP software (SAS Institute, Inc., Cary, NC, USA). *P* values of < 0.05 were considered statistically significant.

## Results and Discussion

### *Oldenlandia diffusa* extract does not inhibit growth of *Haemophilus influenzae*

**Fig 1:**
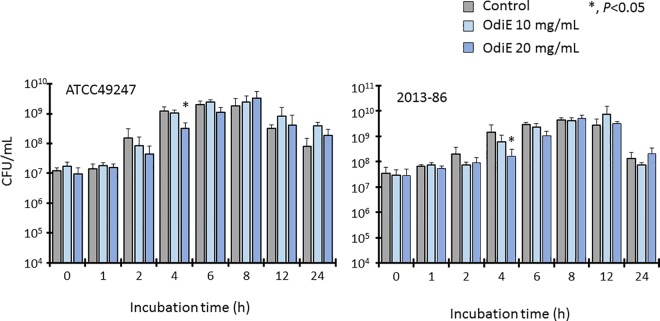
Growth of *H*. *influenzae* with or without *Oldenlandia diffusa* extract. Each experiment was performed three times on separate occasions, and the data are shown as the mean ± standard deviation (SD). The *P* value was calculated by the Welch's *t*-test. OdiE, *Oldenlandia diffusa* extract

To determine whether OdiE could inhibit the growth of *H*. *influenzae*, *H*. *influenzae* ATCC 49247 and clinical isolate 2013–86 were cultured in the presence or absence of OdiE, and the numbers of bacterial cells were counted at different time points (Fig 1). In the presence of 20 mg/mL of OdiE, the number of bacterial cells decreased at 4 h of incubation for both strains, but there were no significant differences with the control after 6 h. Moreover, the numbers of bacterial cells did not decrease in the presence of 10 mg/mL of OdiE. These data suggested that OdiE had a weak antibacterial activity, which was not sufficient to inhibit the growth of *H*. *influenzae*.

### *Oldenlandia diffusa* extract inhibits biofilm formation by *Haemophilus influenzae*

**Fig 2:**
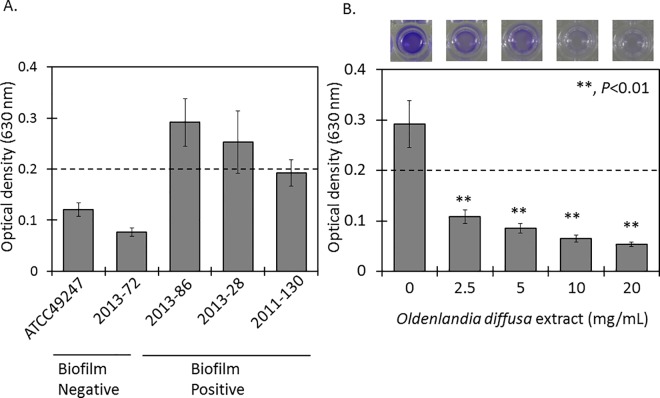
Biofilm formation assay. Biofilm formation was evaluated by the crystal violet assay. (A), Screening for biofilm formation. *H*. *influenzae* ATCC 49247 and 2013–72 represented non-biofilm-forming bacteria. (B), Biofilm formation with or without OdiE. The upper panel shows a photograph of each well after adding 95% ethanol. Each experiment was performed three times on separate occasions, and the data are shown as the mean ± SD. *P* values were calculated by the Student's *t*-test. Dotted line shows the cutoff value for biofilm formation in this study.

**Fig 3:**
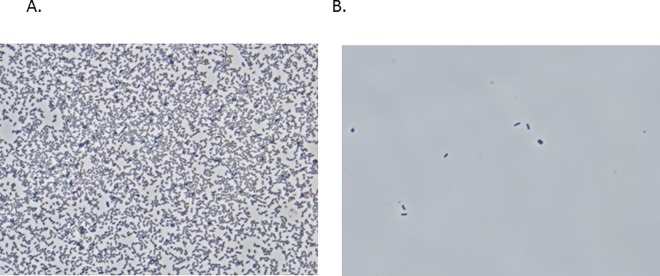
Phase-contrast light microscopic images of *H*. *influenzae* 2013–86 (magnification ×1,000). *H*. *influenzae* 2013–86 was cultured in a 24-well plate, and each well was washed with PBS three times to remove planktonic bacteria. (A) without OdiE (B) with 20 mg/mL of OdiE.

To examine the effect of OdiE on biofilm formation, we first screened the 20 clinical isolates selected for their biofilm formation ability by the crystal violet assay (Data not shown). Based on this screening, two isolates (2013–86 and 2013–28) with a biofilm formation ability and one isolate (2011–130) with a weak biofilm formation ability were selected (Fig 2). All isolates were typed as nontypeable. Two isolates showed susceptibility to β-lactams, macrolides, and levofloxacin, and one isolate showed resistance to β-lactams (Table 1). To test whether OdiE could inhibit biofilm formation, the biofilm amount was measured in the presence or absence of OdiE. Biofilm formation by *H*. *influenzae* 2013–86 was significantly reduced in the presence of OdiE in a concentration-dependent manner (Fig 2, *P* < 0.01). Furthermore, we observed *H*. *influenzae* 2013–86 under a phase-contrast microscope (×1,000) to examine the presence of bacterial cells after washing the wells with PBS. In the presence of 20 mg/mL of OdiE, the number of bacterial cells was clearly smaller than that in the control (Fig 3), indicating that the formation of biofilm was suppressed by the addition of OdiE. Similar results were obtained for the other biofilm-forming *H*. *influenzae* clinical isolates (Table 1).

**Table 1:** Antimicrobial susceptibility and biofilm formation ability of *Haemophilus influenzae* clinical isolates.

Strain	Isolation site	Serotype^a^	Minimum inhibitory concentration (μg/mL)^b^						Biofilm formation (OD_630_) ^c^		*P* value^d^
			Ampicillin	Ampicillin sulbactam	Cefotaxime	Clarithromycin	Azithromycin	Levofloxacin	Without OdiE	With OdiE (20 mg/mL)	
2013–28	Nasal cavity	Nontypeable	0.125	0.125	≤0.063	2	0.25	≤0.063	0.253 ± 0.061	0.096± 0.032	<0.001
2013–86	Nasal cavity	Nontypeable	0.25	0.25	≤0.063	4	0.5	≤0.063	0.292 ± 0.047	0.098 ± 0.032	<0.001
2011–130	Nasal cavity	Nontypeable	8	4	1	2	0.125	≤0.063	0.193 ± 0.025	0.104 ± 0.039	<0.001

^a^Serotype was determined by PCR

^b^minimum inhibitory concentration was measured by the broth dilution method according to the Clinical and Laboratory Standards Institute guidelines

^c^mean ± SD

^d^*P* values were calculated by the Student's *t*-test.

**Fig 4:**
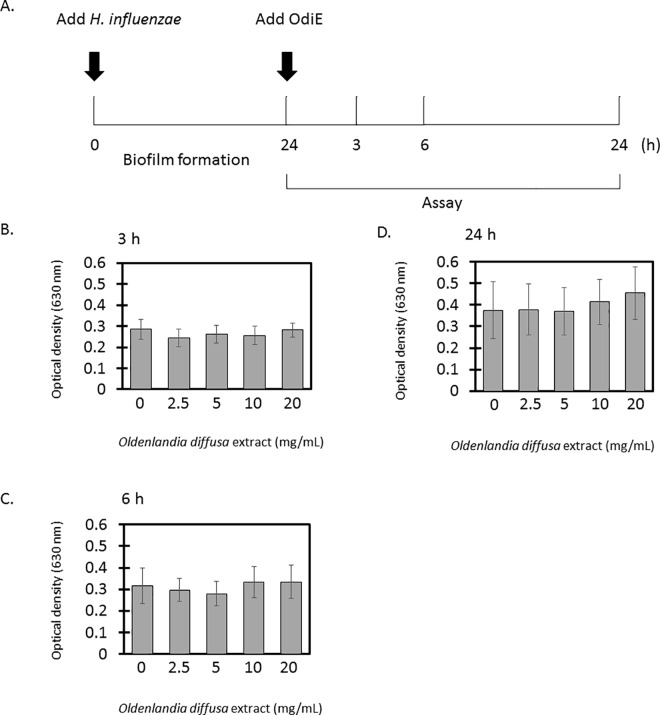
Exfoliative activity of OdiE on biofilm of *H*. *influenzae* 2013–86. (A) analysis scheme (B), 3 h after adding OdiE (C) 6 h after adding OdiE (D) 24 h after adding OdiE. Each experiment was performed three times on separate occasions, and the data are shown as the mean ± SD. The *P* value was calculated by the Student's *t*-test.

To test whether OdiE could exfoliate the formed biofilm, OdiE was added to the formed biofilm, and the amount of biofilm was quantified. The amount of biofilm did not decrease at any concentration of OdiE (Fig 4). These data showed that OdiE could inhibit the formation of biofilm by *H*. *influenzae* but could not exfoliate the formed biofilm.

### *Oldenlandia diffusa* extract inhibits biofilm formation by suppressing LuxS

**Fig 5:**
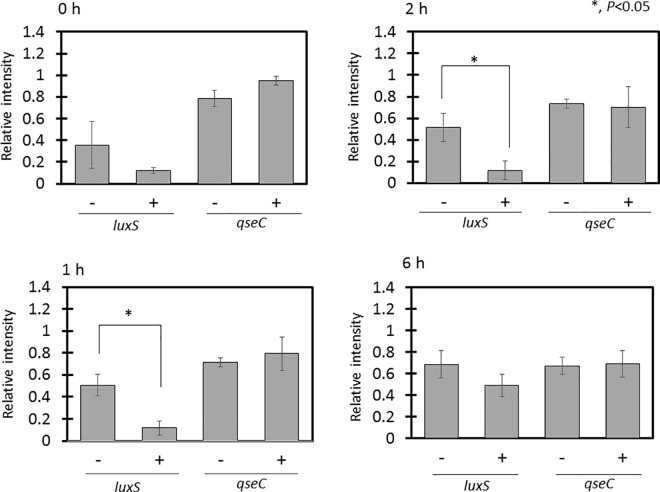
Transcript levels of *H*. *influenzae* 2013–86 biofilm-related genes. Transcript levels of *luxS* and *qseC* were evaluated by semi-quantitative RT–PCR. Relative expression was calculated in comparison with the transcription level of *gyrB*. Each experiment was performed three times on separate occasions, and the data are shown as the mean ± SD. The *P* value was calculated by the Student's *t*-test.

LuxS and the two-component control system QseBC are known to be involved in biofilm formation by *H*. *influenzae* [15]. Therefore, we investigated whether OdiE affects the mRNA levels of these two genes (*luxS* and *qseC*) using RT–PCR. The mRNA level of *luxS* was significantly reduced soon after the OdiE addition, and the suppressive effect continued for at least 2 h, whereas the mRNA level of *qseC* did not change (Fig 5). These results suggested that OdiE inhibits biofilm formation by suppressing its early stage via suppression of *luxS* expression.

**Fig 6:**
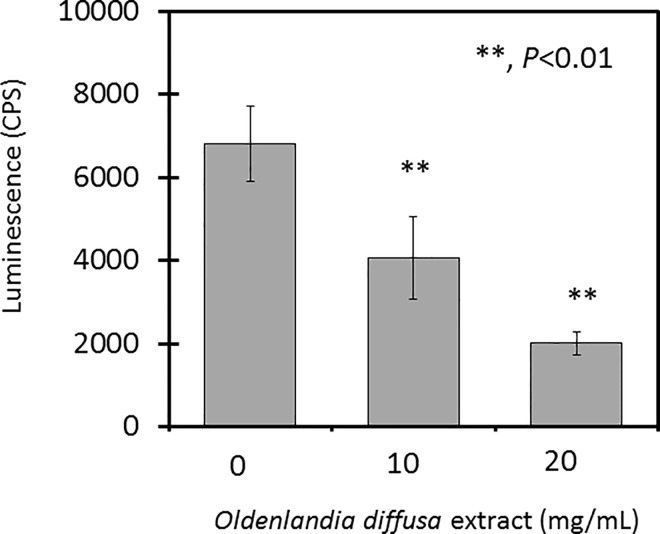
*Oldenlandia diffusa* extract inhibits autoinducer production. The autoinducer in the medium was measured by a bioassay using *Vibrio harveyi* ATCC BAA-211. Each experiment was performed twice on separate occasions, and the data are shown as the mean ± SD. The *P* value was calculated by the Student's *t*-test.

The *luxS* gene is known to be essential for the generation of AI, which is the signal molecule of QS [16]. Since the amount of *luxS* mRNA was reduced by the OdiE addition, we investigated whether the amount of AI was changed using the AI assay with *V*. *harveyi* ATCC BAA-211. By the addition of OdiE, the luminescence was significantly reduced in a dose-dependent manner (Fig 6), indicating that the amount of AI was reduced. Therefore, these data showed that OdiE reduced the secretion of AI by inhibiting the transcription of *luxS* in *H*. *influenzae*, which resulted in a biofilm suppression effect.

Odi has been empirically used as an adjunctive agent for treatments of respiratory infections, such as common cold, for a long time. Interestingly, the unique features revealed in this study, i.e., the OdiE ability to inhibit the biofilm formation but not the growth of *H*. *influenzae*, are consistent with the empirical usage of OdiE. Therefore, our study confirmed the empirical role of OdiE through basic research.

In the bacteriological aspect, agents exhibiting such properties have been rare. In general, a drug that shows biofilm inhibitory effects also shows antimicrobial activity [17], [18]. For example, macrolides are known as antimicrobial agents that also show a biofilm inhibitory activity. In fact, macrolides have been used to treat chronic sinusitis for a long time [19]. The use of antimicrobial agents has been known to lead to an increase of resistant strains by preventing growth of susceptible strains [20]. Actually, macrolide resistance has been increasing not only among *H*. *influenzae* strains but also among *S*. *pneumoniae* and *Mycoplasma pneumoniae* [1]. Since OdiE does not affect bacterial growth, the risk of the emergence of resistant bacteria may be very low.

For an adjunctive therapy agent, its toxicity to humans is a very important factor. A previous study has indicated that OdiE showed toxic effects on cancer cell lines but not on normal cell lines [21], and the IC_50_ values ranged from 7 to 25 mg/mL [21]. Therefore, this strongly suggested that OdiE did not show toxicity for normal cells.

Thus, our data suggested that Odi might have preventive effects on chronic and intractable infections caused by *H*. *influenzae* via prevention of biofilm formation. In the future, it is necessary to analyze the action of Odi against other respiratory pathogenic bacteria, such as *S*. *pneumoniae* and *Moraxella catarrhalis*.

Our study indicated that *O*. *diffusa* could inhibit the biofilm formation by *H*. *influenzae* via suppression of *luxS* transcription, which supports the usefulness of the herb as an adjunctive therapy against respiratory infections.

## References

[1] Morozumi M, Chiba N, Okada T, Sakata H, Matsubara K, Iwata S, et al Antibiotic susceptibility in relation to genotype of *Streptococcus pneumoniae*, *Haemophilus influenzae*, and *Mycoplasma pneumoniae* responsible for community-acquired pneumonia in children. J Infect Chemother. 2013;19(3):432–40. 10.1007/s10156-012-0500-x 23108427

[2] Chinese Materia Medica. Teng J, editor. China: People's Medical Publishing House; 2007.

[3] Song YH, Jeong SJ, Kwon HY, Kim B, Kim SH, Yoo DY. Ursolic acid from *Oldenlandia diffusa* induces apoptosis via activation of caspases and phosphorylation of glycogen synthase kinase 3 beta in SK-OV-3 ovarian cancer cells. Biol Pharm Bull. 2012;35(7):1022–8. 22791147 10.1248/bpb.b110660

[4] Chung HS, Jeong HJ, Hong SH, Kim MS, Kim SJ, Song BK, et al Induction of nitric oxide synthase by *Oldenlandia diffusa* in mouse peritoneal macrophages. Biol Pharm Bull. 2002;25(9):1142–6. 12230105 10.1248/bpb.25.1142

[5] Wiertsema SP, Kirkham LA, Corscadden KJ, Mowe EN, Bowman JM, Jacoby P, et al Predominance of nontypeable *Haemophilus influenzae* in children with otitis media following introduction of a 3+0 pneumococcal conjugate vaccine schedule. Vaccine. 2011;29(32):5163–70. 10.1016/j.vaccine.2011.05.035 21621576

[6] Benninger MS. Acute bacterial rhinosinusitis and otitis media: changes in pathogenicity following widespread use of pneumococcal conjugate vaccine. Otolaryngo Head Neck Surg. 2008;138(3):274–8. 10.1016/j.otohns.2007.11.011 18312870

[7] Murphy TF, Apicella MA. Nontypable *Haemophilus influenzae*: a review of clinical aspects, surface antigens, and the human immune response to infection. Rev Infect Dis. 1987;9(1):1–15. 3547567 10.1093/clinids/9.1.1

[8] Erwin AL, Smith AL. Nontypeable *Haemophilus influenzae*: understanding virulence and commensal behavior. Trends Microbiol. 2007;15(8):355–62. 10.1016/j.tim.2007.06.004 17600718

[9] Novotny LA, Jurcisek JA, Ward MO Jr., Jordan ZB, Goodman SD, Bakaletz LO. Antibodies against the majority subunit of type IV Pili disperse nontypeable *Haemophilus influenzae* biofilms in a LuxS-dependent manner and confer therapeutic resolution of experimental otitis media. Mol Microbiol. 2015;96(2):276–92. PubMed Central PMCID: PMC4423401. 10.1111/mmi.12934 25597921 PMC4423401

[10] Unal CM, Singh B, Fleury C, Singh K, Chavez de Paz L, Svensater G, et al QseC controls biofilm formation of non-typeable *Haemophilus influenzae* in addition to an AI-2-dependent mechanism. Int J Med Microbiol. 2012;302(6):261–9. 10.1016/j.ijmm.2012.07.013 22954413

[11] Wajima T, Seyama S, Nakamura Y, Kashima C, Nakaminami H, Ushio M, et al Prevalence of macrolide-non-susceptible isolates among beta-lactamase-negative ampicillin-resistant *Haemophilus influenzae* in a tertiary care hospital in Japan. J Glob Antimicrob Resist. 2016;6:22–6. 10.1016/j.jgar.2016.01.014 27530834

[12] Vilchez R, Lemme A, Thiel V, Schulz S, Sztajer H, Wagner-Dobler I. Analysing traces of autoinducer-2 requires standardization of the *Vibrio harveyi* bioassay. Anal Bioanal Chem. 2007;387(2):489–96. 10.1007/s00216-006-0824-4 17143597

[13] Puig C, Marti S, Hermans PW, de Jonge MI, Ardanuy C, Linares J, et al Incorporation of phosphorylcholine into the lipooligosaccharide of nontypeable *Haemophilus influenzae* does not correlate with the level of biofilm formation *in vitro*. Infect Immun. 2014;82(4):1591–9. PubMed Central PMCID: PMC3993405. 10.1128/IAI.01445-13 24452688 PMC3993405

[14] Seyama S, Wajima T, Nakaminami H, Noguchi N. Clarithromycin Resistance Mechanisms of Epidemic beta-Lactamase-Nonproducing Ampicillin-Resistant *Haemophilus influenzae* Strains in Japan. Antimicrob Agents Chemother. 2016;60(5):3207–10. PubMed Central PMCID: PMC4862528. 10.1128/AAC.00163-16 26953210 PMC4862528

[15] Armbruster CE, Hong W, Pang B, Dew KE, Juneau RA, Byrd MS, et al LuxS promotes biofilm maturation and persistence of nontypeable *Haemophilus influenzae* in vivo via modulation of lipooligosaccharides on the bacterial surface. Infect Immun. 2009;77(9):4081–91. PubMed Central PMCID: PMC2738029. 10.1128/IAI.00320-09 19564381 PMC2738029

[16] Hardie KR, Heurlier K. Establishing bacterial communities by 'word of mouth': LuxS and autoinducer 2 in biofilm development. Nature Rev Microbiol. 2008;6(8):635–43. 18536728 10.1038/nrmicro1916

[17] Kalia M, Yadav VK, Singh PK, Sharma D, Pandey H, Narvi SS, et al Effect of Cinnamon Oil on Quorum Sensing-Controlled Virulence Factors and Biofilm Formation in *Pseudomonas aeruginosa*. PLoS one. 2015;10(8):e0135495 PubMed Central PMCID: PMC4532483. 10.1371/journal.pone.0135495 26263486 PMC4532483

[18] Sakaue Y, Domon H, Oda M, Takenaka S, Kubo M, Fukuyama Y, et al Anti-biofilm and bactericidal effects of magnolia bark-derived magnolol and honokiol on *Streptococcus mutans*. Microbiol Immunol. 2016;60(1):10–6. 10.1111/1348-0421.12343 26600203

[19] Iino Y, Yoshida N, Kato T, Kakizaki K, Miyazawa T, Kakuta H. Clinical effects of clarithromycin on persistent inflammation following *Haemophilus influenzae*-positive acute otitis media. Acta Otolaryngol. 2015;135(3):217–25. 10.3109/00016489.2014.975893 25649881

[20] Goossens H. Antibiotic consumption and link to resistance. Clin Microbiol Infect. 2009;15 Suppl 3:12–5. 10.1111/j.1469-0691.2009.02725.x 19366364

[21] Gupta S, Zhang D, Yi J, Shao J. Anticancer activities of *Oldenlandia diffusa*. J Herb Pharmacother. 2004;4(1):21–33. 15273074

